# Comparing effects of 4 months of two self-administered exercise training programs on physical performance in patients with chronic kidney disease: RENEXC – A randomized controlled trial

**DOI:** 10.1371/journal.pone.0207349

**Published:** 2018-12-20

**Authors:** Matthias Hellberg, Peter Höglund, Philippa Svensson, Naomi Clyne

**Affiliations:** 1 Department of Nephrology, Institution of Clinical Sciences, Faculty of Medicine, Lund University, Skåne University Hospital, Lund, Sweden; 2 Department of Clinical Chemistry and Pharmacology, Institution of Laboratory Medicine, Faculty of Medicine, Lund University, Skåne University Hospital, Lund, Sweden; University of Bern, SWITZERLAND

## Abstract

**Background:**

Exercise training is recommended to patients with chronic kidney disease (CKD). However, the level of evidence is still low. This randomized controlled trial (RCT) compared two different and self-administered exercise training programs in a representative CKD population.

**Methods:**

This single centre RCT included 151 non-dialysis dependent CKD patients, irrespective of age and comorbidity. Self-administered exercise training of 150 minutes per week was prescribed for 4 months and consisted of 60 minutes endurance training in combination with 90 minutes of either strength or balance training (strength versus balance group). Overall endurance (6-minute walk-test (6-MWT), stair climbing), muscular endurance (30-seconds sit-to-stand (30-STS), heel rises and toe lifts, handgrip (HGS) and isometric quadriceps (IQS) strength, balance (functional reach (FR) and Berg´s balance scale (BBS)) and fine motor skills (Moberg´s picking up test (MPUT)) were measured at baseline and after 4 months. Intention to treat analyses with mixed models was used.

**Results:**

53 women and 98 men, mean age 66 ± 14: range 19 to 87 years, eGFR 20 ± 7: range 8 to 48 ml/min/1.73m^2^ participated. The strength group (n = 76) improved significantly in 6-MWT, stair climbing, 30-STS, heel rises right and left, toe lifts right, IQS right and left, and MPUT with closed eyes with the right and left hand. The balance group (n = 75) improved significantly in heel rises right and left, IQS left, BBS and left-handed MPUT with open and closed eyes. A significant effect between the groups was found for IQS right.

**Conclusions:**

Two different exercise training programs, consisting of endurance in combination with either strength or balance exercise training, improved or maintained overall endurance, muscular strength and endurance, balance and fine motor skills after 4 months of 150 minutes/week self-administered exercise training in a representative CKD population, regardless of age and comorbidity.

## Introduction

Physical inactivity has been identified as the fourth leading risk factor for global mortality causing an estimated 5.2 million deaths annually [1]. At least 30 minutes of moderately intense exercise 5 days per week is recommended by the World Health Organisation for healthy subjects [2].

Patients with chronic kidney disease (CKD) with low self-reported physical activity or low measured physical performance have a higher mortality rate [3–6]. A sedentary lifestyle in CKD patients commonly commences in the early stages of CKD and is an important factor leading to a decrease in physical performance concomitant to the decline in GFR [7, 8]. CKD has systemic effects such as loss of appetite, chronic inflammation, anaemia, metabolic acidosis all contributing to loss of muscle mass and a decline in physical performance. Recent studies have corroborated the systemic effects of CKD on physical performance reporting a deterioration in walking capacity, muscle strength and fatigability, balance and fine motor skills as GFR declines [4, 7, 8].

A Cochrane review highlighting the importance of exercise training in patients with CKD, pointed out the lack of robust evidence and emphasised the need for randomized controlled trials [9]. The Cochrane authors especially advocated RCT´s comparing different exercise training modalities to evaluate the optimal prescription for patients with CKD [9]. To date, most interventional trials in non-dialysis dependent CKD patients comprise small groups of patients, do not include non-dialysis patients at CKD stage 5 and/or have included selected groups of patients [10–16]. There are, to our knowledge, no trials comparing different exercise training modalities.

The aim of this randomized controlled study was to compare the effects of self-administered endurance in combination with either strength or balance exercise training, during 4 months at a moderate intensity for 150 minutes per week, on a broad spectrum of physical performance measures in a representative non-dialysis dependent CKD population, regardless of age and comorbidity. Our hypothesis was that both groups would improve their physical performance measures, but that strength exercise training would be superior to balance exercise training.

## Materials and methods

### Study design

This study was a randomized controlled parallel group interventional single centre trial, conducted between October 2011 and June 2016 at the outpatient clinic of the Department of Nephrology in Lund, Skåne University Hospital. All prevalent and incident CKD patients registered on the outpatient clinic´s uremia list with an eGFR≤30ml/min/1.73m^2^ were invited to participate and were included consecutively. Our goal was to give all CKD patients interested in the RENEXC trial the opportunity to participate if there were no contraindications or potential risks for patients´ health. All adults 18 years or older with any comorbid burden were accepted. A patient was excluded if he/she showed one or more of the following conditions: severe orthopaedic or neurologic disorders, unstable cardiovascular disease, uncontrolled hypertension, severe anaemia, severe electrolyte disturbances, inability to communicate in Swedish or to understand oral instructions or was expected to start renal replacement therapy within one year after recruitment.

After baseline assessment and examination, the participants were randomized into two exercise training groups and re-assessed after 4 months’ intervention. The strength group was prescribed endurance and strength exercise training and the balance group endurance and balance exercise training.

#### Power calculation

To detect a difference of at least 60% of the standard deviation at a two-sided 5% significance level and 80% power, we calculated to include 50 patients in each group. In order to compensate for a dropout rate of about 30% we decided to include at least 75 patients in each group.

#### Ethics approval and trial registration

The study was approved by the Regional Ethical Review Board in Lund (registration number 2011/369) on August 9, 2011, prior to recruitment and adhered to the Helsinki Declaration. All participants gave informed written consent prior to inclusion after having received written and oral information.

The study is part of the RENEXC trial and registered as NCT02041156 at www.ClinicalTrials.gov.

The registration process started on October 10, 2011 after approval by the Regional Ethical Review Board. The first patient was recruited on October 31, 2011 and the last patient on January 19, 2016. The last patient performed the 4 months assessment on June 8, 2016. The reason for the delayed official confirmation from www.ClinicalTrials.com was because of changes in the administrative staffs’ responsibilities, due to the merger of two university hospitals into one. These circumstances were out of the investigators control. The authors confirm that all ongoing and related trials for this intervention are registered.

#### Ethical considerations

As exercise training is recommended by the Swedish Nephrological Society and the Swedish Kidney Patients Association as well as being an integrated part of usual care at our department, it was not practically or ethically feasible to have a non-exercising control group. Thus, the study was designed to comprise two treatment arms.

Physical performance was assessed by a dedicated research physiotherapist, who also prescribed the individual exercise training for each patient.

### Randomization

After inclusion and baseline assessment the patients were randomly assigned to the strength or balance group by a computer generated random allocation (SAS Proc Plan, SAS Institute Cary NC). Recruiting staff, including each patient’s doctor, was blinded to randomization. Only the research physiotherapist and the patients themselves were aware of which exercise group they belonged to.

### Exercise training program

Each patient was provided with an individual exercise training program based on his/her physical performance assessment results at baseline and according to randomization. The goal was to perform a total of 150 minutes of exercise training, distributed between 3 to 5 sessions per week, at a submaximal exertion level for 4 months. The weekly training included 60 minutes of endurance and 90 minutes of either strength or balance training. The training was prescribed by the physiotherapist from a bank of exercises and was self-administered by the patients at home or at a nearby gym, according to the patient’s preference. Each training session was preceded by a warm-up.

### Training follow-up

Each patient was advised how to evaluate the intensity of his/her exercise training by using the Borg Scale of Rate of Perceived Exertion (RPE) [17]. The patients were asked to keep training diaries. The physiotherapist could be reached every workday in case of questions. During the first 3 months, the physiotherapist telephoned each patient every week and then every second week to check progress, to encourage patients and to adjust the training to maintain the RPE constant. Both groups had the same schedule for follow-ups, assessments and received the same amount of attention from the physiotherapist. Thus, guaranteeing the similarity of both the intervention arms.

The patients were asked to report exercise training related side effects, unintended effects or harm.

### Endurance training

The endurance training was part of the training program in both groups. The goal for the duration of the endurance training was to reach a minimum of 60 minutes per week (30 minutes x 2). The aim for the intensity was to reach a submaximal exertion defined as 13–15 RPE i.e. ‘somewhat strenuous’ to ‘strenuous’. Activities that were prescribed included walking, jogging, cycling, cross-trainer, stair climbing etc. Intensity was raised by increasing the speed, the duration or performing interval training.

### Strength training

Strength training focused on larger muscle groups with sessions of 30 minutes 3 times per week. Patients were asked to perform 4–6 different exercises per session, consisting of 2 sets of 10 repetitions. The aim was to reach a submaximal exertion defined as 13–17 RPE i.e. ‘somewhat strenuous’ to ‘very strenuous’. The resistance was gradually augmented by using heavier weights or increasing the difficulty of bodyweight exercises (by adjusting the body position regarding angle or leverage). Exercises were performed in open or closed kinetic chains depending on the individual and the environment in which the training was performed. Examples for prescribed exercises were quadriceps extensions, hamstrings curls, latissimus pull-downs, squats, push ups, biceps curls etc.

### Balance training

Balance training focused on balance and stability with sessions of 30 minutes 3 times per week. Exercises holding a static position (e.g. standing with the feet together, standing on one leg, standing on a balance board, tandem stance or planking) were performed at 3–5 sets of 30–60 seconds. The aim was to reach a submaximal exertion defined as 13–17 RPE i.e. ‘somewhat strenuous’ to ‘very strenuous’. The difficulty was gradually increased by adding arm movements, closing eyes or changing body position. Dynamic balance exercises such as lunges with upper body rotation or single-leg dead-lift were performed at 2–3 sets of 10 repetitions. For both static and dynamic balance exercises an unstable surface, such as an exercise ball, was used to activate further core stability and increase difficulty.

### Physical performance measures—Primary outcomes

Each patient was assessed prior to start and after 4 months of exercise training. All physical performance tests have previously been presented in detail [4, 7] and comprise:

- **Overall endurance** (walking and climbing capacity) with 6-minute walk-test (6-MWT) and stair climbing
- **Muscular endurance and fatigability** in the proximal muscles with chair-rises (30-STS, 30-seconds sit-to-stand) and in the distal muscles with heel rises and toe lifts right and left
- **Neuromuscular function and strength** in the lower extremities with right and left isometric quadriceps strength and in the upper extremities with right and left handgrip strength
- **Balance** with functional reach and Berg´s balance scale
- **Fine motor skills** by Moberg´s picking up test left- and right handed with open and with closed eyes

There were no changes in trial outcomes after the trial commenced.

### Training intensity and adherence–secondary outcomes

The duration of each session, specified as time spent on endurance, strength or balance exercises respectively, and the achieved RPE level for each exercise were registered by each patient in their training diaries and reported weekly for registration in the data base.

### Clinical chemistry and estimated GFR

Laboratory analyses were measured at the Department of Clinical Chemistry, Laboratory Medicine Skåne, which is accredited by SWEDAC (Swedish Board of Accreditation and Conformity Assessment) according to the international standards ISO 15189:2012, using routine methods at baseline and after 4 months. Glomerular filtration rate was estimated (eGFR) by using the cystatin C- and creatinine-based GFR-prediction equation [18].

### Comorbidity

The comorbid burden was recorded using the Davies Comorbidity Score [19] at the time of visit and physical examination at the outpatient clinic prior to inclusion by MH. The Davies Comorbidity Score consists of 7 possible comorbid domains, comprising of malignancy, diabetes mellitus, left ventricular dysfunction, ischemic heart disease, peripheral vascular disease, systemic collagen vascular disease and other diseases with impact on survival.

### Statistical methods

Continuous variables are presented as means ± standard deviations or 1^st^ - 2^nd^ - 3^rd^ quartiles. Categorical variables are given as percentages and frequencies. Intention to treat analyses was used to compare the two exercise groups and all randomized patients were included. To address missing data, we chose the mixed model analysis and the results of the physical performance tests at baseline and at 4 months were evaluated using the following effects: time, treatment and their interaction as fixed effects and subjects as random effects. We used an unstructured covariance model in these analyses. Effects are presented as estimates and with 95% confidence intervals. Data were analysed using the R software (www.r-project.org).

Data from the RENEXC trial on the patients´ clinical characteristics at baseline have been presented previously in a cross-sectional baseline study [20]. To facilitate understanding of the prospective results from the randomized trial, RENEXC, patients´ clinical characteristics at baseline data are also given in this paper.

## Results

**Fig 1:**
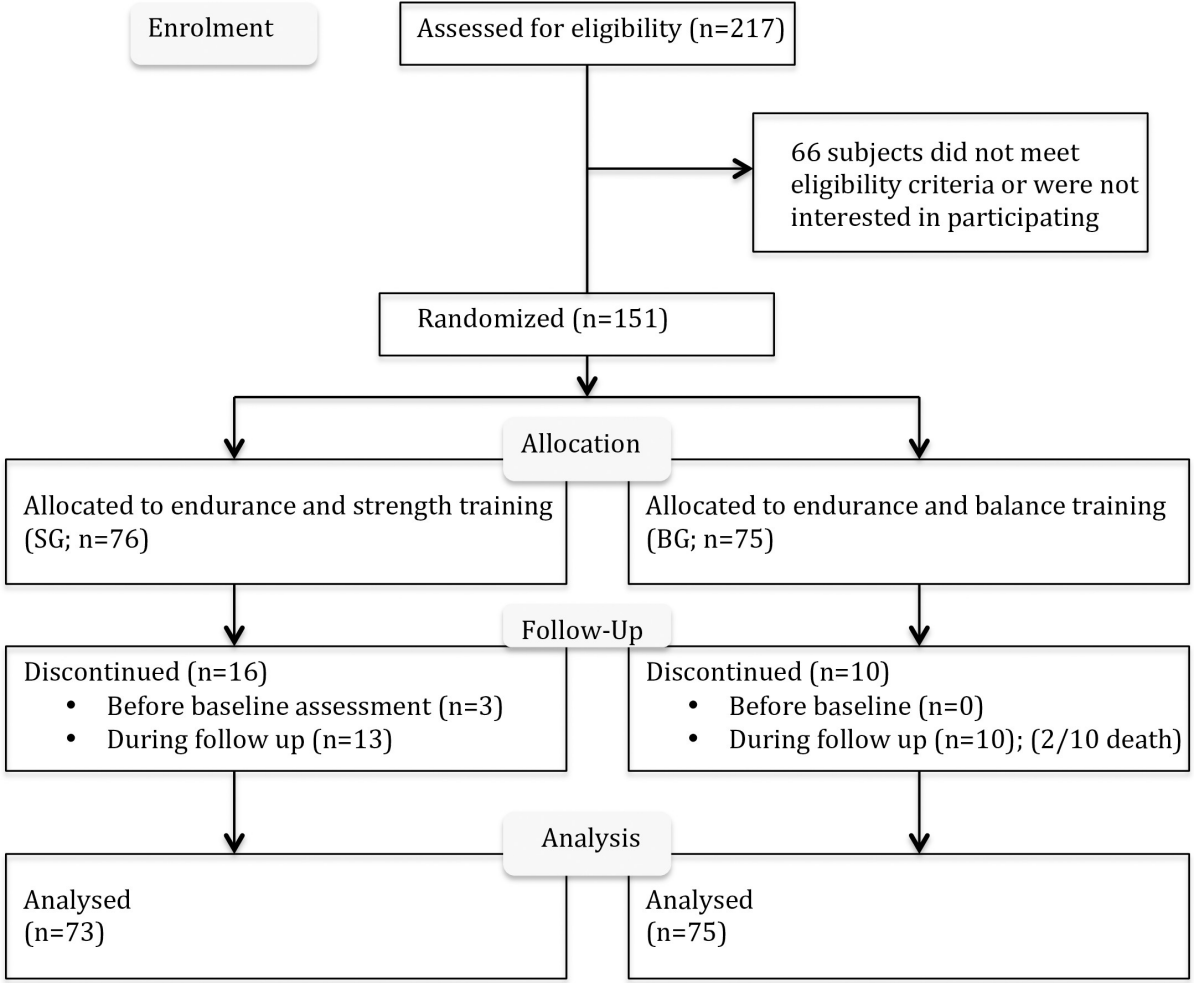
CONSORT flow diagram—RENEXC.

We screened 217 patients with eGFR ≤30ml/min/1,73m^2^ of whom 151 were included (53 women and 98 men) with a mean age of 66 ± 14: range 19 to 87 years. The causes of kidney disease were hypertension (41%; n = 62), diabetes mellitus (16%; n = 24), glomerulonephritis (15%; n = 23), interstitial nephritis (15%; n = 22), polycystic kidney disease (6%; n = 9) and others (7%; n = 11). Three patients dropped out shortly after randomization and before baseline assessment due to bone fracture, terminal illness and recurring retinal haemorrhage. The CONSORT diagram (Fig 1) shows that 13 patients in the strength group and 10 in the balance group discontinued during follow-up. Two patients died, for reasons unrelated to the study. The other reasons for the lack of data after 4 months are concomitant illness, travel, moved to another town or not motivated to continue.

The clinical characteristics (Table 1) and the physical performance measures (Table 2) are presented for the whole study group at baseline.

**Table 1:** General clinical characteristics in the strength and balance group.

	Units	N	Strength group	N	Balance group
Sex	male/female	76	25/51	75	28/47
Age	years	76	67±14	75	65±14
BMI	kg/m^2^	73	28±6	75	27±5
eGFR	ml/min/1.73m^2^	72	19±8	74	20±7
P-cystatin C	mg/L	72	2.9±0.7	72	2.8±0.7
P-creatinine	μmol/L	73	263±102	73	246±106
P-urea	mmol/L	73	16±5	75	16±6
CRP	mg/L	73	6±7	75	5±9
P-albumin	g/L	73	37±4	75	37±3
Hemoglobin	g/L	73	126±15	74	128±14
Base excess	mmol/L	68	-1.3±3.3	74	-1.2±2.9
P-potassium	mmol/L	71	4.3±0.5	75	4.1±0.5
P-calcium	mmol/L	73	2.3±0.1	74	2.3±0.2
P-phosphate	mmol/L	73	1.1±0.3	75	1.2±0.3
PTH	pmol/L	72	11 (8, 21)	73	13 (10, 18)
U-ACR	mg/mmol	70	18 (4, 127)	74	29 (4, 109)
24-hour ambulatory blood pressure (systolic/diastolic)	mmHg (day)	67	133/78±15/11	72	134/79±16/10
	mmHg (night)	67	120/66±19/9	69	121/68±18/11
**Comorbidity**					
Malignancy		12	16%	9	12%
Ischemic heart disease		17	22%	13	17%
Peripheral vascular disease		18	24%	13	17%
Left ventricular dysfunction		6	8%	10	13%
Diabetes mellitus		29	38%	27	27%
Systemic collagen vascular disease		9	12%	7	9%
Other significant pathology (e.g. hypertension)		58	76%	57	76%
**Medication**					
Antihypertensive medication		72	95%	71	95%
Beta blockade		51	67%	50	67%
RAAS blockade		48	63%	43	57%
Central antiadrenergic medication		6	8%	10	13%

Mean ± standard deviation; median (1^st^Q, 3^rd^Q), Q = quartiles; BMI = body mass index

eGFR = estimated GFR = Cystatin-C and creatinine based

P = plasma; CRP = C-reactive protein, PTH = parathyroid hormone

U-ACR = urine-albumin-creatinine-ratio; RAAS = renin-angiotensin-aldosterone-system.

**Table 2:** Physical performance in the whole study population at baseline.

				Units		N	Observed values	Relative values
**Endurance (overall)**								
	Walking capacity		6-MWT	m		147	402±137	78±23
	Climbing capacity			n		147	7 (4,13)	NA
**Muscular endurance and fatigability**								
	Proximal leg muscles		30-STS	n		147	11±16	72±36
	Distal leg muscles		Heel rises	n	right	142	7 (0, 20)	28 (0, 80)
				n	left	143	7 (0, 21)	28 (0, 80)
			Toe lifts	n	right	142	2 (0, 15)	10 (0, 75)
				n	left	143	2 (0, 11)	10 (0, 53)
**Neuromuscular exercise function/strength**								
	Lower extremity							
		Isometric quadriceps strength		kg*m	right	144	11.4±4.1	91±26
				kg*m	left	144	11.3±4.2	90±26
	Upper extremity							
		Handgrip strength		kg	right	148	32±10	84±21
				kg	left	147	28±11	84±23
**Balance**								
	Functional reach			cm		146	32±10	97±24
	Berg balance scale		score	n		145	51±8	NA
**Fine motor skills**								
	Moberg’s picking up test							
		With open eyes		sec	right	146	8.3±2.1	NA
				sec	left	145	8.5±2.4	NA
		With closed eyes		sec	right	146	23.0±13.9	NA
				sec	left	145	23.2±8.9	NA

Mean ± standard deviation; median (1^st^Q, 3^rd^Q), Q = quartiles; NA = not available; relative values in %, related to the expected norm; 6-MWT = 6-minute walk test; 30-STS = 30-seconds sit to stand.

### Strength group—Effects on physical performance

In the strength group the following aspects of physical performance increased: overall endurance, measured with the 6-MWT by 4% and stair climbing by 20%, muscular endurance in the proximal muscles measured with 30-STS by 9% and in the distal muscles measured with heel rises right by 17% and left by 27% and toe lifts right by 29%, muscular strength in the legs measured with quadriceps strength right by 11% and left by 8% and fine motor skills with closed eyes in both hands measured with Moberg´s picking up test right by 13% and left by 6%. Table 3

**Table 3:** Strength group: Effects of 4 months of endurance and strength training on physical performance.

						Values at baseline			Values after 4 months			Effects after 4 months	
				Units		N	Observed	Relative	N	Observed	Relative	Mean observed	Mean relative
**Endurance (overall)**													
	Walking capacity		6-MWT	m		73	379±138	75±25	60	400±149	79±29	14 [0.3–28]	3 [0.2–5]
	Climbing capacity			n		72	6 (4, 12)	NA	60	8 (5, 14)	NA	2 [0.3–3]	NA
**Muscular endurance and fatigability**													
	Proximal leg muscles		30-STS	n		73	11±6	64±36	59	11±7	69±41	1 [0.2–1]	5 [1–9]
	Distal leg muscles		Heel rises	n	right	70	7 (0, 20)	28 (0, 80)	59	11 (0, 22)	44 (0, 88)	2 [0.1–4]	7 [0.3–14]
				n	left	71	7 (0, 20)	28 (0, 80)	59	13 (0, 27)	52 (0, 106)	3 [0.5–5]	10 [2–18]
			Toe lifts	n	right	69	0 (0, 13)	0 (0, 65)	58	4 (0, 19)	18 (0, 96)	2 [1–4]	12 [3–22]
				n	left	70	1 (0, 10)	5 (0, 50)	59	4 (0, 14)	20 (0, 68)	2 [0–4]	9 [0–18]
**Neuromuscular exercise function/strength**													
	Lower extremity												
		Isometric quadriceps strength		kg*m	right	71	11.5±4.1	91±28	58	12.8±4.2	101±29	1.3 [0.8–1.7]	10 [7–14]
				kg*m	left	71	11.6±4.3	92±27	58	12.6±4.7	99±32	0.9 [0.3–1.4]	7 [3–11]
	Upper extremity												
		Handgrip strength		kg	right	73	32±10	84±19	59	33±9	86±18	0 [-1-1]	0 [-2-2]
				kg	left	73	30±10	84±21	59	30±9	84±20	0 [-1-1]	0 [-2-3]
**Balance**													
	Functional reach			cm		72	32±9	95±24	60	33±9	96±26	1 [0–3]	4 [0–9]
	Berg balance scale			score		72	50±9	NA	60	51±9	NA	0.6 [-0.1–1.4]	NA
**Fine motor skills**													
	Moberg’s picking up test												
		With open eyes		sec	right	73	8.6±2.3	NA	60	8.3±2.2	NA	-0.4 [-0.7–0.0]	NA
				sec	left	73	8.6±2.7	NA	60	8.4±2.6	NA	-0.3 [-0.7–0.0]	NA
		With closed eyes		sec	right	73	24.3±18.2	NA	60	21.4±8.7	NA	-3.1 [-5.6-(-0.3)]	NA
				sec	left	73	23.3±8.9	NA	60	22.0±8.5	NA	-1.5 [-2.9-(-0.1)]	NA

Mean ± standard deviation; median (1^st^Q, 3^rd^Q), Q = quartiles; NA = not available; [..-..] = 95% confidence interval; relative values in % and related to reference values, related to the expected norm; 6-MWT = 6-minute walk test; 30-STS = 30-seconds sit to stand.

### Balance group—Effects on physical performance

In the balance group the following aspects of physical performance increased: muscular endurance in the distal muscles measured with heel rises right by 25% and left by 25%, muscular strength in the legs measured with quadriceps strength left by 6%, balance measured with Berg´s balance scale by 2% and fine motor skills measured with Moberg´s picking up test in the left hand with open eyes by 5% and with closed eyes by 9%. Table 4

**Table 4:** Balance group: Effects of 4 months of endurance and balance training on physical performance.

						Values at baseline			Values after 4 months			Effects after 4 months	
				Units		N	Observed	Relative	N	Observed	Relative	Mean observed	Mean relative
**Endurance (overall)**													
	Walking capacity		6-MWT	m		74	425±133	82±21	63	445±147	85±23	11 [-3-25]	2 [0–5]
	Climbing capacity			n		75	7 (5, 13)	NA	59	7 (5, 15)	NA	0 [-1-2]	NA
**Muscular endurance and fatigability**													
	Proximal leg muscles		30-STS	n		74	12±6	73±36	65	13±6	75±34	1 [0–1]	3 [-1-6]
	Distal leg muscles		Heel rises	n	right	72	9 (0, 21)	34 (0, 84)	61	15 (2–27)	60 (8, 108)	3 [1–5]	12 [5–18]
				n	left	72	8 (0, 21)	32 (0, 85)	61	11 (0–28)	44 (0, 112)	3 [1–5]	12 [4–20]
			Toe lifts	n	right	73	3 (0, 16)	15 (0, 80)	60	7 (0–19)	33 (0, 96)	1 [0–3]	7 [-2-16]
				n	left	73	3 (0, 15)	15 (0, 75)	61	5 (0–18)	25 (0, 90)	2 [0–3]	8 [-1-17]
**Neuromuscular exercise function/strength**													
	Lower extremity												
		Isometric quadriceps strength		kg*m	right	73	11.4±4.1	91±25	62	11.7±4.3	92±26	0.0 [-0.4–0.5]	0 [-4-4]
				kg*m	left	73	11.1±4.2	88±24	62	11.9±4.5	94±27	0.7 [0.2–1.2]	6 [2–10]
	Upper extremity												
		Handgrip strength		kg	right	75	31±11	84±22	64	32±12	87±24	0 [-1-1]	0 [-2-2]
				kg	left	74	29±11	83±24	63	29±11	85±27	0 [-1-1]	0 [-3-2]
**Balance**													
	Functional reach			cm		74	34±9	95±25	63	35±8	103±22	1 [-1-2]	3 [-2-7]
	Berg balance scale			score		73	52±6	NA	65	53±5	NA	1 [0.02–1]	NA
**Fine motor skills**													
	Moberg’s picking up test												
		With open eyes		sec	right	73	8.0±2.0	NA	64	7.8±2.0	NA	-0.1 [-0.5–0.3]	NA
				sec	left	72	8.3±1.9	NA	64	7.8±1.9	NA	-0.4 [-0.7-(-0.1)]	NA
		With closed eyes		sec	right	73	21.7±7.3	NA	64	20.8±7.7	NA	-0.8 [-3.5–1.9]	NA
				sec	left	72	23.1±9.0	NA	64	20.7±8.2	NA	-2.0 [-3.4-(-0.7)]	NA

Mean ± standard deviation; median (1^st^Q, 3^rd^Q), Q = quartiles; NA = not available; [..-..] = 95% confidence interval; relative values in % and related to reference values, related to the expected norm; 6-MWT = 6-minute walk test; 30-STS = 30-seconds sit to stand.

### Between group effects for the strength group compared with the balance group

Apart from a statistically significant greater increase in quadriceps strength right in the strength group (P<0.001), there were no statistically significant differences between groups. Table 5

**Table 5:** Differences in physical performance between the strength- and the balance group after 4 months of intervention.

				Δ estimated effects after 4 months of training			
				Units		Absolute	Relative
**Endurance (overall)**							
	Walking capacity		6-MWT	m		3 [-16-23]	1 [0–5]
	Climbing capacity			n		2 [-1-4]	NA
**Muscular endurance and fatigability**							
	Proximal leg muscles		30-STS	n		0 [-1-1]	2 [-3-8]
	Distal leg muscles		Heel rises	n	right	-1 [-4-1]	-4 [-14-5]
				n	left	0 [-3-2]	-2 [-13-10]
			Toe lifts	n	right	1 [-2-4]	5 [-8-19]
				n	left	0 [-2-3]	1 [-12-14]
**Neuromuscular exercise function/strength**							
	Lower extremity						
		Isometric quadriceps strength		kg*m	right	1.3 [0.6–1.9]	10 [5–16]
				kg*m	left	0.1 [-0.6–0.8]	1 [-4-7]
	Upper extremity						
		Handgrip strength		kg	right	0 [-1-1]	0 [-3-3]
				kg	left	0 [-1-1]	0 [-3-4]
**Balance**							
	Functional reach			cm		1 [-2-3]	2 [-5-8]
	Berg balance scale		score	n		0 [-1-1]	NA
**Fine motor skills**							
	Moberg’s picking up test						
		With open eyes		sec	right	-0.2 [-0.8–0.3]	NA
				sec	left	0.1 [-0.4–0.6]	NA
		With closed eyes		sec	right	-2.3 [-6.2–1.6]	NA
				sec	left	0.5 [-1.4–2.4]	NA

Δ estimated effects = differences of the estimated effects by mixed model analysis in the strength group compared with the balance group; NA = not available; [..-..] = 95% confidence interval; relative values in %, related to the expected norm; 6-MWT = 6-minute walk test; 30-STS = 30-seconds sit to stand.

### Strength group—Training adherence and intensity

The strength group reported 88–144–183 (1^st^– 2^nd^– 3^rd^) minutes per week of total training time, i.e. endurance and strength. Endurance training was performed for 33–64–99 minutes per week. The total number of exercise sessions was 19–33–51. The RPE was 10–13–17. The strength training was performed for 43–69–97 minutes per week. The number of strength exercise sessions was 26–39–59 sessions. The RPE was 8–13–14. The cumulative time of endurance training was 8.2–14.7–26.8 hours. The cumulative time of strength training was 10.3–19.1–27.5 hours.

### Balance group—Training adherence and intensity

The balance group reported 80–116–173 minutes per week of total training time, i.e. endurance and balance. Endurance training was performed for 38–73–108 minutes per week. The total number of endurance exercise sessions was 28–40–56. The RPE was 11–12–13. The balance training was performed for 28–46–67 minutes per week. The total number of balance exercise sessions was 21–38–54. The RPE was 9–12–14. The cumulative time of endurance training time was 11.2–20.0–31.4 hours. The cumulative time of balance training was 7.4–12.6–18.7 hours.

Neither the strength- nor the balance group showed any significant association between training duration, level of exertion and effects on physical performance.

No exercise training related side effects, unintended effects or harm were reported by the participants to any of the study personal during the intervention period in either group.

## Discussion

### Effects of 4 months of self-administered exercise training

Both the strength and the balance group improved or stabilised all measures of physical performance after 4 months of exercise training. No group showed deterioration in any of the tests. Significant improvements in both groups could be found in muscular endurance and fatigability in the distal leg muscles, in muscular quadriceps strength and fine motor skills. A number of exercise training studies have shown improvement in VO_2peak_, 6-MWT, sit-to-stand tests and various measures of muscle strength. Unique for our study is the comprehensive battery of tests for a broad spectrum of different forms of physical performance. To our knowledge, this is the first study in which fine motor skills were tested and showed an improvement after exercise training. This could have important implications in the management of a number of aspects of activities of daily living.

Furthermore, the strength group improved walking distance and climbing capacity as well as muscular endurance and fatigability in the proximal leg muscles.

The balance group improved balance evaluated by the Berg´s balance scale, but not measured with functional reach. In a previous baseline study from the RENEXC trial we found an association between balance measured with Berg´s balance scale and lean mass in the trunk and balance measured with functional reach and lean mass in the legs [20]. In line with these findings, the balance group in the present study showed a significant improvement of quadriceps strength in the left leg, but not in the dominant right leg and showed no improvement in muscle fatigability in the proximal leg muscles, which could explain why there was no improvement in functional reach. The improvement in balance is of clinical relevance as loss of balance corresponds to a high risk of falls and a high mortality risk especially in the dialysis population [21, 22].

Even though the strength group showed significant improvements in more of the tests than the balance group, the only significant between group effect was found for quadriceps strength in the right leg in favour of the strength group. The prevalence of right handedness and right footedness, was 90% in a subgroup of our study population, which is in accordance with the general population. This could possibly elucidate why the strength group showed a between group effect in favour of the right quadriceps muscle.

### Adherence to 4 months of 150 minutes of weekly exercise training

The high acceptance of the patients screened (71%) showed patients´ interest and motivation to exercise irrespective of age and comorbidity, thus affirming the feasibility of our broad inclusion criteria. The completion rate was also high (83%) as was adherence to the exercise prescription of 150 minutes per week: on average 98% in the strength group and 107% in the balance group. This is impressive for such a large cohort of patients, comprising elderly and frail patients, conducting home- or local gym based training. It is in line with the cohort of 36 stage 3–4 CKD patients in the LANDMARK 3 study with a similar adherence [23], but in contrast to another study with a decrease in adherence from 80 to 20% when switching from centre- to home based training in a cohort of 10 patients [24]. There were several important contributing factors to the high adherence in our study: 1. the individual adaptation of the exercise prescription to each patient’s physical performance at baseline, 2. the use of the rate of perceived exertion to monitor exercise training intensity, 3. the regular telephone calls from the physiotherapist and 4. her high accessibility, support and encouragement. An earlier study showed that regular telephone contact per se resulted in better outcomes of self-administered exercise training compared with standard contact [25].

### Home- or centre-based exercise training

In our study patients had the option to train at home or at a nearby gym. The majority of the patients chose to train at home. A previous study in hemodialysis patients reported the best outcome but highest withdrawal rate, after 6 months of exercise training, for a supervised in-centre training group on non-dialysis days compared with a group training during dialysis and a group with self-administered on non-dialysis days [26]. The largest randomized controlled exercise training trial in CKD patients to date, the EXCITE study, was conducted in dialysis patients who were prescribed a simple home-based walking exercise program managed by dialysis staff with a completion rate of nearly 70% in the exercise training arm and an adherence rate of 83% [27]. The dialysis staff in the EXCITE study encouraged patients to adhere to the exercise training and provided feedback in conjunction with each dialysis session [27]. The EXCITE trial resulted in improved walking distance and quality of life [27]. In an exploratory uncontrolled feasibility study with predialysis, dialysis and renal transplant patients, comprising a twice-weekly supervised outpatient exercise training and education session run by physiotherapists and a once-weekly home-based exercise training session for a period of 12 weeks, 59% (77 of 131) patients included completed the training period [28].

Investigations in exercise training physiology have shown that there is no linear relationship between training duration, level of exertion and the effects on physical performance. The lower the level of physical performance at baseline, the greater the effect that can be achieved by intervention [29]. Furthermore, the results also depend on each patient’s motivation and especially on each patient’s specific goals. The goals can vary but could for example be to be able to walk to the nearby store without having to take a break or visit a grandchild who lives in an apartment on the third floor without a lift.

### RENEXC–largest RCT in a representative non-dialysis dependent CKD population

The RENEXC study is to our knowledge the largest randomized controlled exercise training trial in patients with non-dialysis dependent CKD comparing different exercise training modalities.

Most previous exercise training studies in non-dialysis dependent CKD patients have lower numbers of participants, who often are highly selected, have few comorbidities and usually better renal function [10–16]. The RENEXC-study was designed with wide inclusion criteria so that elderly patients and patients with a high comorbid burden could participate. Our intent was to conduct a randomized controlled trial with the exercise training integrated into everyday clinical practice, so that the results could be applicable outside the structure of a clinical trial. Our findings are representative for the typical CKD patient and are therefore easily generalizable. This practical approach was also adopted in the choice of tests used to evaluate physical performance. They should be easy to apply in the regular clinical setting and not require expensive equipment. The tests were easily comprehendible within the context of the patient’s everyday life like being able to go shopping, to climb stairs or keep one’s balance and avoid falls.

### Strengths and limitations of the study

There are many studies both in healthy elderly people and in patients with CKD showing that at least three months of regular exercise training is usually required for improvement, which is why we chose a 4 months intervention period. An intervention period of more than 4 months would have provided stronger evidence on the long-term feasibility of self-administered exercise training. As this study had broad inclusion criteria comprising patients with non-dialysis dependent CKD stages 3 to 5, of all ages, including elderly and very elderly patients and with a number of comorbidities, we chose a 4 months’ intervention period. Thus, we cannot comment on the long-term evolution of these exercise training programmes on the patients’ physical performance. In this study we did not study possible benefits of exercise training on daily life or clinical status. It is not unreasonable to deduct that improved physical performance at the least makes patients more able to cope with their everyday life. A long time follow up of our training concept would be motivated and benefits for daily life should be addressed.

By using intention to treat and mixed model analyses, we could make as realistic and honest an analysis as possible of all randomized study patients. Study information, medical history and physical examination of each patient were performed by the same physician (MH). There was only one change of physiotherapist, which occurred after the first study year, after which the same physiotherapist tested all the patients, prescribed the exercise training and was responsible for follow-up. Each patient received the same attention and procedure, thus avoiding differences between the two training groups, especially with regards to motivation.

Instead of a non-exercising control group, we chose two treatment arms in analogy with pharmacological studies with active controls. Our hypothesis was that endurance combined with strength exercise training would be more efficacious in increasing physical performance than endurance in combination with balance exercise training. However, a study with a sedentary control group would probably have achieved larger group effects and thus our study design can be viewed as a potential weakness.

Physiotherapists are part of renal care units in Sweden. At our clinic in Lund all patients with eGFR of about 30 ml/min/1,73m^2^ or less are offered a comprehensive investigation program. Assessment of physical performance and recommendations for exercise training are part of it. Exercise training is part of our department´s official policy and accepted in routine clinical practice. By having two treatment arms, we eliminated the ethical dilemma of a sedentary control group. The two treatment arms assured that both groups had the same opportunity to adhere to their respective treatment. In this context, the risk that a sedentary control group would receive recommendations by other physicians and staff to exercise was avoided. The single-centre design could also be regarded as a limitation. The follow up method for compliance is a weakness as it relies on self-reporting and regular phone calls. However, the phone calls are also a strength of the study as an important aim was to motivate the patients and quickly discover problems they might have.

To date, there are an increasing number of devices, which can be used for registration of physical activity like activity bands, pedometers or apps on smart phones and others. At the time the study was planned and started such devices were not as common as today and still quite expensive. An exercise training study starting today should and could easily integrate such a part of activity registration in the follow-up, which would make registration of actually performed physical activity easier than self-reported training diaries.

### Conclusion

Two different exercise training programs, consisting of endurance in combination with either strength or balance exercise training, improved or maintained overall endurance, muscular strength and endurance, balance and fine motor skills after 4 months of 150 minutes/week self-administered exercise training in a representative non-dialysis dependent CKD population, regardless of age and comorbidity. Thus, the combination of endurance with either strength or balance exercise training is feasible in clinical routine and effectively maintains or increases physical performance.

Of interest is whether these training results can be maintained or further improved over a longer period, as well as possible effects on kidney function and other factors, which will be investigated in further studies.

## Supporting information

S1 File CONSORT checklist. (DOCX)

S2 File Research program. (DOCX)
