# Supplementary material for: Comparing effects of 4 months of two self-administered exercise training programs on physical performance in patients with chronic kidney disease: RENEXC – A randomized controlled trial
Source: PLoS One. 2018 Dec 20;13(12):e0207349. doi: 10.1371/journal.pone.0207349 (PMC6301660; doi:10.1371/journal.pone.0207349)
Supplement: S1 File — (DOCX) [file pone.0207349.s001.docx]

## S1 File. CONSORT checklist

| **Section/** | **Item** |  |  |  |  |  |  |  | **Reported** |
| --- | --- | --- | --- | --- | --- | --- | --- | --- | --- |
| **Topic** | **No** |  |  |  |  |  |  |  | **on page No** |
| **Title and abstract** | |  |  |  |  |  |  |  |  |
|  | **1a** | **Identification as randomised trial in the title** | | | |  |  |  | **1** |
|  | **1b** | **Structured summary of trial design, methods, results, and conclusions** | | | | | |  | **2-3** |
| **Introduction** |  |  |  |  |  |  |  |  |  |
|  | **2a** | **Scientific background and explanation of rationale** | | | | |  |  | **3-4** |
|  | **2b** | **Specific objectives or hypothesis** | | |  |  |  |  | **3-4** |
| **Methods** |  |  |  |  |  |  |  |  |  |
|  | **3a** | **Description of trial design (such as parallel, factorial) including allocation ratio** | | | | | | | **4-5** |
|  | **3b** | **Important changes to methods after trial commencement (such as eligibility criteria),** | | | | | | | **5** |
|  |  | **with reasons** | |  |  |  |  |  |  |
| **Participants** |  |  |  |  |  |  |  |  |  |
|  | **4a** | **Eligibility criteria for participants** | | |  |  |  |  | **5-6** |
|  | **4b** | **Settings and locations where the data were collected** | | | | |  |  | **5-6** |
| **Interventions** |  |  |  |  |  |  |  |  |  |
|  | **5** | **The interventions for each group with sufficient details to allow replication, including** | | | | | | | |
|  |  | **how and when they were actually administered** | | | |  |  |  | **6-8** |
| **Outcomes** |  |  |  |  |  |  |  |  |  |
|  | **6a** | **Completely defined pre-specified primary and secondary outcome measures,** | | | | | | |  |
|  |  | **including how and when they were assessed** | | | |  |  |  | **8-9** |
|  | **6b** | **Any changes to trial outcomes after the trial commenced, with reasons** | | | | | |  | **8** |
| **Sample size** |  |  |  |  |  |  |  |  |  |
|  | **7a** | **How was sample size determined** | | |  |  |  |  | **5** |
|  | **7b** | **When applicable, explanation of any interim analyses and stopping guidelines** | | | | | | | **none** |
| **Randomisation** | |  |  |  |  |  |  |  |  |
| **Sequence generation** | |  |  |  |  |  |  |  |  |
|  | **8a** | **Method used to generate the random allocation sequence** | | | | |  |  | **6** |
|  | **8b** | **Type of randomisation; details of any restriction (such as blocking and block size)** | | | | | | | **6** |
| **Allocation concealment mechanism** | | |  |  |  |  |  |  |  |
|  | **9** | **Mechanism used to implement the random allocation sequence (such as sequentially** | | | | | | | |
|  |  | **numbered containers), describing any steps to conceal the sequences until** | | | | | | |  |
|  |  | **interventions were assigned** | | |  |  |  |  | **6** |
| **Implementation** |  |  |  |  |  |  |  |  |  |
|  | **10** | **Who generated the random allocation sequence, who enrolled participants,** | | | | | | |  |
|  |  | **and who assigned participants to interventions** | | | |  |  |  | **6** |
| **Blinding** |  |  |  |  |  |  |  |  |  |
|  | **11a** | **If done, who was blinded after assignment to interventions** | | | | |  |  |  |
|  |  | **(for example, participants, care providers, those assessing outcomes) and how** | | | | | | | **6** |
|  | **11b** | **If relevant, description of the similarity of interventions** | | | | |  |  | **6** |
| **Statistical methods** | |  |  |  |  |  |  |  |  |
|  | **12a** | **Statistical methods used to compare groups for primary and secondary outcomes** | | | | | | | **9-10** |
|  | **12b** | **Methods for additional analyses, such as subgroup analyses and adjusted analyses** | | | | | | | **none** |
| **Results** |  |  |  |  |  |  |  |  |  |
| **Participant flow (a diagram is strongly recommended)** | | | | |  |  |  |  |  |
|  | **13a** | **For each group, the numbers of participants who were randomly assigned,** | | | | | | |  |
|  |  | **received intended treatment, and were analysed for the primary outcome** | | | | | |  | **Fig 1** |
|  | **13b** | **For each group , losses and exclusions after randomisation, together with reasons** | | | | | | | **10** |
| **Recruitment** |  |  |  |  |  |  |  |  |  |
|  | **14a** | **Dates defining the periods of recruitment and follow-up** | | | | |  |  | **5** |
|  | **14b** | **Why the trial ended or was stopped** | | |  |  |  |  | **5** |
| **Baseline data** |  |  |  |  |  |  |  |  |  |
|  | **15** | **A table showing baseline demographic and clinical characteristics for each group** | | | | | | | **Tables 1 and 2** |
| **Numbers analysed** | |  |  |  |  |  |  |  |  |
|  | **16** | **For each group, number of participants (denominator) included in each analysis and** | | | | | | | |
|  |  | **whether the analysis was by original assigned groups** | | | | |  |  | **Tables 3 and 4, Fig 1** |
| **Outcomes and estimation** | | |  |  |  |  |  |  |  |
|  | **17a** | **For each primary and secondary outcome, results for each group,** | | | | | |  |  |
|  |  | **and the estimated effects size and its precision (such as 95% confidence interval)** | | | | | | | **10-17** |
|  | **17b** | **For binary outcomes, presentation of both absolute and** | | | | |  |  |  |
|  |  | **relative effect sizes is recommended** | | |  |  |  |  | **10-17** |
| **Ancillary analyses** | |  |  |  |  |  |  |  |  |
|  | **18** | **Results of any other analyses performed, including subgroup analyses and** | | | | | | |  |
|  |  | **adjusted analyses, distinguishing pre-specified from exploratory** | | | | | |  | **none** |
| **Harms** |  |  |  |  |  |  |  |  |  |
|  | **19** | **All important harms or unintended effects in each group** | | | | |  |  |  |
|  |  | **(for specific guidance see CONSORT for harms)** | | | |  |  |  | **17** |
| **Discussion** |  |  |  |  |  |  |  |  |  |
| **Limitations** |  |  |  |  |  |  |  |  |  |
|  | **20** | **Trial limitations, addressing sources of potential bias, imprecision, and,** | | | | | |  |  |
|  |  | **if relevant, multiplicity of analyses** | | |  |  |  |  | **21-22** |
| **Generalisability** | |  |  |  |  |  |  |  |  |
|  | **21** | **Generalisability (external validity, applicability) of the trial findings** | | | | | |  | **17-22** |
| **Interpretation** | |  |  |  |  |  |  |  |  |
|  | **22** | **Interpretation consistent with results, balancing benefits and harms, and** | | | | | |  |  |
|  |  | **considering other relevant evidence** | | |  |  |  |  | **17-22** |
| **Other information** | |  |  |  |  |  |  |  |  |
| **Registration** |  |  |  |  |  |  |  |  |  |
|  | **23** | **Registration number and name of trial** | | | |  |  |  | **5** |
| **Protocol** |  |  |  |  |  |  |  |  |  |
|  | **24** | **Where the full trial can be accessed, if available** | | | |  |  |  | **5** |
| **Funding** |  |  |  |  |  |  |  |  | **entered in sub-** |
|  | **25** | **Sources of funding and other support (such as supply of drugs), role of funders** | | | | | | | **mission system** |
