# Supplementary material for: Comparing effects of 4 months of two self-administered exercise training programs on physical performance in patients with chronic kidney disease: RENEXC – A randomized controlled trial
Source: PLoS One. 2018 Dec 20;13(12):e0207349. doi: 10.1371/journal.pone.0207349 (PMC6301660; doi:10.1371/journal.pone.0207349)
Supplement: S2 File — (DOCX) [file pone.0207349.s002.docx]

# Research program: Effects of exercise training in chronic kidney disease (CKD)

## Specific objectives:

Investigating the effects of regular exercise training for 150 minutes per week in patients with CKD regardless of age and comorbidity in a prospective randomized controlled trial (RCT) by comparing the effects of two different exercise training programs focusing on endurance exercise training in combination with either strength or balance exercise training

1. Does regular endurance and strength exercise training have different effects on physical performance and the status in CKD patients than regular endurance and balance exercise training? Are there differences after 4, 8 and 12 months of exercise training intervention? What can be concluded?
2. Can regular exercise training performed as two different exercise training programs, i.e. endurance combined with either strength or balance exercise training, affect the progress of decline in kidney function in CKD patients?
3. Do data analyses support the hypothesis of better prognosis regarding morbidity and mortality in CKD patients by regular exercise training?

## Previously published results:

Patients with CKD have an increased mortality in cardiovascular disease and infections compared to the general population. Remarkably, the risk is not age-related as in the normal population, but 20 to 30 year-old hemodialysis patients showed the same risk of cardiovascular mortality as 80 years olds in the general population according to data from the US dialysis database [1]. The level of physical activity is a well-known and important factor for survival in the general population [2, 3]. In recent years, there are several studies showing that CKD patients do not constitute an exception [4, 5]. Self-perceived level of physical activity is often low in CKD patients especially when starting chronic hemodialysis; approximately 60% report no physical activity per week at all [6]. This group of patients with very low level of physical activity also showed a poorer survival during a 5 years period compared with patients with higher physical activity [6]. Aerobic capacity, measured with VO_2peak_, had a strong predictive value for survival in dialysis patients, where a group of VO_2peak_ <17.5 ml/min/kg had 19 deaths in 88 patients while the group with VO_2peak_ >17.5 ml/min/kg had 4 deaths in 87 patients [4]. In this study, inactivity had the same detrimental effect on survival as malnutrition.

The natural course in CKD patients shows a progressive deterioration of maximal exercise capacity with increased renal impairment [7], with maximal exercise capacity, measured with a cycle ergometer, was approximately 50 to 60% of the expected norm in patients on chronic dialysis treatment [8].

Uremia is a pronounced catabolic disease and already in CKD stage 3 muscle atrophy begins to occur [9], which gradually increases with decreasing renal function. In patients on hemodialysis, the muscular endurance of the thigh muscles was at a level corresponding to 25% of age and sex matched reference values [10].

In hemodialysis patients, regular and rather intense aerobic exercise during a 9 months period resulted in a decrease in left ventricular mass index, an increase in cardiac output and ejection fraction, and an increased heart rate index and lower arrhythmia rate [11, 12]. Blood pressure control improved after 4 months of exercise training in a controlled trial with predialysis patients [13]. A group of dialysis patients could maintain good blood pressure control after 6 months of regular exercise training while reducing the number of antihypertensive drugs [14]. Twelve months of exercise training with a cycle ergometer resulted in significantly improved levels of blood lipids, faster glucose elimination and higher insulin sensitivity in a controlled trial with patients on dialysis [15]. Quality of life has shown significant improvements after exercise training in patients on dialysis, with decreased symptoms of depression and higher levels of self-perceived physical performance and bodily pain in the SF-36 questionnaire [16, 17, 18, 19].

Experimental studies with uremic rats have shown that exercise training slows the progression of uremia [20]. A recently published clinical trial has shown that a higher level of physical activity had a beneficial effect on the progression of kidney function in the elderly, where patients with high physical activity had an almost 30% lower risk of rapid renal impairment defined as loss of GFR of >3ml/min/1.73m^2^ per year [21].

The uremic syndrome is strongly catabolic, experimental studies in both rats and humans show there is a loss of amino acids due to outflow from uremic muscle due to proteolysis [22, 23]. The reason of this proteolysis is multifaceted and dependent on metabolic acidosis through the ubiquitin proteasome pathway, insulin resistance, cytokine mediated inflammation with activation of C-reactive Protein (CRP), interleukin-6 (IL-6), interleukin-1β (IL-1β) and tumour necrosis factor α (TNF α), malnutrition and physical inactivity. In patients with CKD stage 5, muscle fibre atrophy [24] and reduced muscle mass measured as lean body mass [25] as well as changed muscle fibre composition were correlated to a decline in kidney function [24].

Human studies during a hemodialysis session have shown that insulin resistance is an important factor affecting muscle wasting even in patients who do not have diabetes mellitus [26]. Insulin resistance, measured by the homeostatic assessment index (= (the product of solid insulin and fasting glucose) divided by 22.5) correlated to net protein loss with increasing insulin resistance [26]. Patients with diabetes mellitus showed significantly higher net protein loss compared to patients without diabetes during hemodialysis [27].

Uremic rats could slow this increased muscle catabolism by intensive aerobic exercise training in form of daily swimming [22]. They did not increase the inflow of amino acids and thus protein synthesis, but reduced the outflow, thus achieving a balance between the in- and outflow of amino acids across muscle cell membranes. A recently published experimental study on mice showed that aerobic exercise reduced the outflow of amino acids from skeletal muscle, but did not increase the inflow, but strength exercise training in contrast led to increased protein synthesis in the muscle [28]. Recently published studies showed an increase in the net balance of protein metabolism in the skeletal muscle in hemodialysis patients who trained during dialysis session and were supplemented with both oral and parenteral nutrition during dialysis [29, 30]. Strength exercise training in patients with CKD stage 4 to 5 caused muscle hypertrophy and decreased inflammation with significantly lower level of CRP and IL-6 and higher level of transferrin [31], also hemodialysis patients demonstrated lower levels of CRP after strength exercise training [32].

## Working plan

A prospective randomized controlled interventional trial will be performed to investigate the effects of regular exercise training in patients with CKD at the outpatient clinic of the Department of Nephrology at the University Hospital in Lund and is planned to start in autumn 2011. All incident and prevalent CKD patients on the uremia list will be invited to take part. The participants will be randomized to two different but standardized and individualized exercise training programs consisting of endurance exercise training combined with either strength exercise training or balance exercise training.

Today, in clinical routine, all patients with CKD stage 4 and 5 are offered to receive a standardized package of investigations including contact establishment with the renal care staff (renal failure nurse, physiotherapist, dietician and social worker) called the uremia assessment. All incident and prevalent CKD patients are registered in the clinics` uremia list as well as in the Swedish National Register (SNR) for CKD.

Based on the established routines of the usual uremia assessment some further investigations will be added and instead of a general exercise training recommendation by the physiotherapist each patient will receive a prescription of exercise training according to randomization. Each patient receives usual renal failure care at the outpatient clinic regardless of participation in the trial. The routine follow-up uremia reassessment after one year will be performed as usual by each patients´ nephrologist, but will be strictly structured and protocolled according to the trial requirements. A dedicated research physiotherapist is necessary and will be employed to provide the extended physical performance assessments, the individualized exercise training program for each patient as well as to be available for the participants during follow-up.

Parts of the randomized controlled trial will be part of the nephrologist´s Matthias Hellberg´s doctoral dissertation. All results and analyses are intended to be published and thus made publicly available. In addition, all participants will be invited to annual information events for updates on the trial progress as well as results.

## Study design

The study will be a consecutive, prospective randomized controlled trial. All prevalent and incident patients on the uremia list at the outpatient clinic at the Nephrology Department in Lund will be invited to participate regardless of age and comorbidity. All CKD patients with GFR <30 ml/min/1,73m^2^ will be offered the uremia assessment and will be registered on the uremia list and thus invited to participate in the trial. CKD patients younger than 18 years are followed up at the department of pediatrics and will not be offered participation. If patients´ status should constitute a contraindication for exercise training or would involve a potential risk to the patients´ health the patient will not be included. Exclusion criteria will be unstable angina pectoris, uncontrolled hypertension, uncompensated heart failure, muscle disease, orthopedic or neurological disorders or diseases relevant for the completion of the trial.

The intervention will consist of 150 minutes per week of exercise training for 12 months. Two different exercise training modalities will be compared and thus the patients will be randomized to either the strength group or the balance group. Patients in the strength group will be prescribed 60 minutes per week endurance exercise training combined with 90 minutes per week strength exercise training. The balance group will be prescribed 60 minutes per week endurance exercise training combined with 90 minutes per week balance exercise training. Extended uremia assessment will be performed at baseline, after 4, 8 and 12 months of the intervention period and thereafter as the usual annually uremia assessment follow-up.

## Statistical background

150 patients will be needed to be included in the study, 75 in each exercise training group. We calculated to find differences at the level of 60% of one standard deviation at a 5% level of significance and 80% strength if we accrued complete data on 50 patients in each group. Intention to treat and mixed model analyses will be used. All the physical performance measurements will be the primary outcomes of the trial. All the other uremia assessment results of the patients´ status will be secondary outcomes of the trial.

## Method

The nephrologist Matthias Hellberg is the project manager and will be responsible for screening of all prevalent and incident CKD patients on the uremia list as well as providing each potential participant with written and oral information about the trial. He will be responsible that each patient will have given informed consent prior to inclusion. He will meet each patient at the outpatient clinic for physical examination prior to inclusion, and will register comorbidity by using Davies´ comorbidity score and register the current medication of each participant.

After inclusion each patient will be provided with an extended uremia assessment, coordinated by the renal failure nurses and the research physiotherapist as well as the medical secretary. The extended uremia assessment will take two workdays as well as the usual uremia assessment.

Each patient is provided with the usual uremia assessment at baseline and after 12 months of the exercise training intervention period, consisting of: iohexol clearance, heart and lung x-ray, ECG (electrocardiogram), echocardiography, DEXA (dual-energy x-ray absorptiometry) with measurement of bone density and body composition, BMI (body mass index) as well as the socioeconomic status by the social worker and the dietary counselling by the dietician. This annual usual uremia assessment will be supplemented by the measurement of AAC (abdominal aorta calcification) and SGA (subjective global assessment by the dietician).

The measurement of physical performance is also part of the annual usual uremia assessment with stair climbing, heel rises, toe lifts, isometric quadriceps strength, handgrip strength, functional reach and Moberg´s picking up test. This will be supplemented with 6-minute walk test, 30-seconds sit to stand and Berg´s balance scale. The assessment of the extended physical performance measurements will be performed at baseline and after 4-, 8- and 12 months of the intervention period.

Blood samples as part of the usual uremia assessment for analysing of electrolyte-, blood-, iron-, liver-, lipid-, thyroid-, acid- and base-, calcium- and phosphate status as well glucose and HbA1c, PTH, urate, cobalamin, folate, CRP and albumin will be supplemented with one extra blood sample of 2 ml for later analysis of inflammatory markers. All these blood samples will be collected at baseline and after 4, 8 and 12 months of the intervention period. Urine samples with urine-albumin/creatinine ratio will be supplemented with 24-hours urine-urea and will be measured at baseline, after 4, 8 and 12 months as well.

Further additions to the usual uremia assessments will be the measurements of 24 hours blood pressure, 24 hours ECG and a questionnaire for evaluating the quality of life in uremic patients (KDQol-SF^TM^); all these will be provided at baseline, after 4, 8 and 12 months.

Apart from the measurement of AAC and the questionnaire (KDQol-SF^TM^) all investigations and assessments are part of established clinical routines at the University Hospital in Lund.

The abdominal aorta calcification (AAC) will be scored by the renal failure nurses from the lateral x-ray image over the lumbar spine. The questionnaire (KDQol-SF^TM^) will be provided by the research physiotherapist on the physical performance assessment days.

The project manager will be responsible for any referrals and will sign all the results collected during the assessments. If abnormal results occur the project manager will be responsible for taking the necessary actions.

After baseline assessment a computer generated randomization program will allocate each patient to either the strength or balance group. Only the research physiotherapist and each patient will be informed about the group affiliation. The research physiotherapist will prescribe thereafter the individualized exercise training program for each patient based on the patients´ assessed physical performance results and according to the randomization. The exercise training can be provided at home or at a nearby gym. Participants who chose the gym will be reimbursed the costs for the membership at the gym.

The research physiotherapist will prepare a bank of exercises for the three exercise training modalities (endurance, strength and balance), from which exercises can be selected to ensure comparable and as similar as possible prescriptions for the exercise training. Each patient will be trained by the research physiotherapist to evaluate the intensity of the exercise training by using the Borg scale of Received Perceived Exhaustion (RPE). The RPE level should range between 13 to 17 of maximum 20 to achieve moderate intense exercise training.

The exercise training will be prescribed as 150 minutes per week with 60 minutes of endurance exercise training and 90 minutes of either strength or balance exercise training. A warm-up will be preceded every exercise training session. The exercise training time can be spent between 3 to 5 times per week.

Training diaries will be prepared by the project manager in collaboration with the research physiotherapist and each patient will be asked to record training time and intensity. The patients will be asked to send the training diaries to the outpatient clinic for registration in the data base by the research physiotherapist. The patients will also be asked to report exercise training related side effects, unintended effects or harm during the trial.

The exercise training program for each patient will be self-administered at home or a nearby gym. The research physiotherapist will call each patient weekly during the first 3 months and second weekly during 4 to 12 month of the intervention period to check the progress, to encourage and to adjust the exercise training to achieve training intensity.

Endurance exercise training will be prescribed as 60 minutes per week and can be distributed on 2 occasions with 30 minutes and should achieve 13 to 15 of RPE.

Strength exercise training will be prescribed as 90 minutes per week and can be distributed on 3 occasions with 30 minutes and should achieve 13 to 17 of RPE.

Balance exercise training will be prescribed as 90 minutes per week and can be distributed on 3 occasions with 30 minutes and should achieve 13 to 17 of RPE.

The clinic has two regular physiotherapists and these will be well informed about the trial to assist in the case of absence of the research physiotherapist.

The uremia assessment, the exercise training prescription of 150 minutes per week consisting of 60 minutes of endurance and 90 minutes of other (strength or balance) exercise training as well as the same follow-up procedure will guarantee the same attention to each participant regardless of affiliation to the strength or the balance group.

After 12 months of exercise training intervention the patients will be followed up with annual usual uremia assessments according to standard procedure.

All results of the uremia assessments as well as patients´ morbidity and hospitalization will be recorded in the hospital´s electronic journal data base and from there they will be transferred to a coded excel file for analyses and evaluation. The transfer code key will be kept locked in a metal cabinet. Only the project manager will have access. The material will be coded and stored as long as the trial and the analyses will be on-going.

## Clinical importance

CKD patients are suffering from a chronic toxic syndrome that affects all organs of the body. Increased proteolysis with muscle wasting and impaired physical performance are important symptoms in advanced CKD. A low-grade chronic inflammation contributes to muscle wasting and is also considered to be a contributing factor to the high cardiovascular morbidity of this patient group described as the MIA syndrome (malnutrition-inflammation-atherosclerosis-syndrome). In short-term studies, regular exercise training has been shown to increase both overall endurance as well as muscular endurance and strength in patients with CKD and showed positive effects on some of uremia´s medical complications. Most of these studies have been conducted in patients already on dialysis treatment, where the deleterious effects of uremia have already been established. It is important to investigate whether previous interventions can influence the development of uremic complications and which type of exercise is most beneficial.

There are currently no long-term studies investigating the effects of exercise training on patients with CKD stage 4 to 5. Some of the positive exercise training effects on cardiovascular disease occur only after about one year of regular exercise training, so it is important to carry out long-term follow-up of the effects of regular exercise training on patients with CKD stage 4-5. Furthermore, these patients eventually become dependent on advanced life-sustaining dialysis treatment and exercise training gives the patients the opportunity to take the command over part of their live and treatment and gives a strong sense of empowerment.

Equally important for the patients’ self-esteem and immediate well-being are the effects of exercise training on ability to cope with everyday physical requirements and stresses as well as the effects on patient´s mental status and quality of life. Exercise training as supplementary treatment may not have only positive effects for the individual patient but also potentially positive socioeconomic consequences in the way that more patients can manage themselves at home, perform peritoneal dialysis at home by themselves or drive a car to the dialysis centre by themselves instead of wheelchair or stretcher transportation.

Effects of exercise training on physical performance are expected within 4 to 8 months as well as reduced inflammatory activity. Long-term effects with, for example, improved level of blood pressure, lipidstatus or glucose tolerance are expected at the earliest after 12 month of exercise intervention.

Two different training modalities will be compared. If different exercise training modalities, i.e. endurance combined with either strength or balance exercise training, have different effects in CKD patients will be investigated by the trial. Any differences will be analysed in and may give the opportunity to optimize the recommendation of exercise training in CKD in the future and will increase the level of evidence for exercise training in CKD and thus will create the prerequisite for a possible future prescription of exercise training. The beneficial effect of regular exercise training on the progress of decline in kidney function in rats has so far not been shown in humans, but will evaluated after 12 month of exercise training intervention due to the analysis of measured GFR by iohexol clearance, assessed at baseline and after 12 months.

Long-term effects such as prolonged uremia progress, later onset of active renal replacement therapy, less morbidity and mortality in cardiovascular disease are not expected to be found within the 12 months of exercise training intervention period, but will be registered as morbidity and hospitalisation during long-term follow-up and annual usual uremia assessments, even after start in renal replacement therapy up to the end of their lives.

## Scientific importance

There are still relatively few prospective randomized controlled trials published that provide a high level of evidence for recommendation of exercise training in CKD patients. However, the trials are still too few with too small numbers of participants in order to achieve a level of evidence that would be necessary to offer exercise training as a supplementary treatment in CKD. In addition to this, there is still a lack of evidence for which training modality is most appropriate and should be recommended or prescribed to CKD patients. The long-term effects of regular exercise training will be evaluated and may answer questions related to regular exercise training and morbidity and or mortality in CKD.

The present study will address these issues and thus, the trial is important to perform.

References
1.Foley RN, Parfrey PS, Sarnak MJ. Clinical epidemiology of cardiovascular disease in chronic renal failure. Am J of Kidn Dis 1998(32)5, suppl 3:S112-S119.
2.Sandvik L, Erikssen J, Thaulow E et al. Physical fitness as a predictor of mortality among healthy middle-aged Norwegian men. NEJM 1993(328):533-7.
3.Paffenbarger RS, Hyde RT, Wing AL et al. The association of changes in physical activity level and other lifestyle characteristics with mortality in men. NEJM, 1993(328):538-45.
4.Sietsema KE, Amato A, Adler SG et al. Exercise capacity as a predictor of survival among ambulatory patients with end-stage renal disease. Kidn Int, 2004(65):719-724.
5.O’Hare AM, Tawney K, Bacchetti P et al. Decrease survival among sedentary patients undergoing dialysis: Results from the dialysis morbidity and mortality study wave 2. Am J Kidn Dis, 2003(41):447-454.
6.Stack AG, Molony DA, Rives T et al. Association of physical activity with mortality in the US dialysis population. Am J Kidn Dis, 2005(45):690-701.
7.Clyne N, Jogestrand T, Lins LE et al. Progressive decline in renal function induces a gradual decrease in total hemoglobin and exercise capacity. Nephron, 1994(67):322-326.
8.Painter P, Messer-Rehak D, Hanson P et al. Exercise capacity in hemodialysis, CAPD and renal transplant patients. Nephron, 1986(41):47-51.
9.Foley RN, Wang C, Ishani A et al. Kidney function and sarcopenia in the United States general population: NHANES III. Am J Nephrol, 2007(27)3:279-286.
10.Sandström, A. Jämförelse mellan statisk och dynamisk muskeluthållighet hos hemodialyspatienter och ett friskt åldersmatchat referensmaterial. Kandidatuppsats, Karolinska institutet, 1997.
11.Deligiannis A, Koudi E, Tassoulas E et al. Cardiac effects of exercise rehabilitation in hemodialysis patients. In J Cardiol, 1999(70):253-266.
12.Deligiannis A, Kouidi E, Tourkantonis A. Effects of physical training on heart rate variability in patients on hemodialysis. Am J Cardiol, 1999(84):197-202.
13.Boyce ML, Robergs RA, Avasthu PS et al. Exercise training by individuals with predialysis renal failure: Cardiorespiratory endurance, hypertension, and renal function. Am J of Kidn Dis, 1997(30):2:180-192
14.Miller BW, Cress CL, Johnson ME et al. Exercise during hemodialysis decreases the use of antihypertensive medications. Am J of Kidney Diseases, 2002(39)4:828-833.
15.Goldberg AP, Hagberg J, Delmez JA et al. The metabolic and psychological effects of exercise training in hemodialysis patients. The Am J of Clin Nutr,1980(33): 1620-1628.
16.Carney RM, Templeton B, Hong BA et al. Exercise training reduces depression and increases the performance of pleasant activities in hemodialysis patients. Nephron, 1987(47):194-198.
17.Kouidi E, Iacovides A, Iordanidis P et al. Exercise renal rehabilitation program: Psychosocial effects. Nephron, 1997(77):152-158.
18.Painter P, Carlson L, Carey S et al. Physical functioning and health-related quality-of-life changes with exercise training in hemodialysis patients. A Journal of Kidney Diseases, 2000(35)3:482-492.
19.Molsted S, Eidemak I, Sorenson HT et al. Five months of physical exercise in hemodialysis patients: Effects on aerobic capacity, physical function and self-rated health. Nephron Clin Pract, 2004(96):c76-c81.
20.Osato S, Onoyama K, Okuda S et al. Effect of swimming exercise on the progress of renal dysfunction in rat with focal glomerulosclerosis. Nephron, 1990(55):306-311.
21.Robinson-Cohen C, Katz R, Mozaffarian D et al. Physical activity and rapid decline in kidney function among older adults. Arch Int Med 2009(169):22:2116-23.
22.Davies TA, Karl IE, Goldberg AP et al. Effects of exercise training on muscle protein catabolism in uremia. Kidn Int, 1983(24), suppl16:S52-S57.
23.Garibotto G, Russo R, Sofia a, et al. Skeletal muscle protein synthesis and degradation in patients with chronic renal failure. Kidn Int, 1994(45):1432-1439.
24.Clyne N, Esbjörnsson M, Jansson E, Jogestrand T, Lins LE, Pehrsson SK. Effects of Renal Failure on Skeletal Muscle. Nephron 1993;63:395-399.
25.Pupim LB, Heimbürger O, Qureshi AR, Ikizler TA, Stenvinkel P. Accelerated lean body mass loss in incident chronic patients with diabetes mellitus. Kidn Int 2005(68:5:2368-74.
26.Siew ED, Pupim LB,Shintani A, et al. Insulin resistance is associated with skeletal muscle protein breakdown in non-diabetic chronic hemodialysis patients. Kidn Int 2007(71):146-152.
27.Lee WL, Park GH, Lee SW, Song JH, Hong KC, Kim MJ. Insulin resistance and muscle wasitning in non-diabetic end-stage renal disease patients. Nephrol Dial transplant 2007; 22:2554-62.
28.Wang XH, Jie D, Klein JD, Bailey JL, Mitch WE. Exercise ameliorates chronic kidney disease–induced defects in muscle protein metabolism and progenitor cell function. Kidn Int 2009; 76(7):751-760.
29.Majchrzak K, Pupim L, Flakoll, et al. Resistance exercise augments the acute anabolic effects of intradialytic oral nutritional supplementation. Nephrol Dial Transplant, 2007(23:1362-1369.
30.Pupim LB, Flakoll PJ, Levenhagen DK et al. Exercise augments the acute anabolic effects of intradialytic parenteral nutrition in chronic hemodialysis patients, Am J Physiol Endocrinol Metab, 2004(286):E589-E597.
31.Castaneda C, Gordon PL, Parker RC et al. resistance training to reduce the malnutrition-inflammation complex syndrome of chronic kidney disease. Am J of Kidn Dis, 2004(43):4:607-616.
32.Cheema B, Abas H, Smith B et al. Progressive exercise for anabolism in kidney disease (PEAK): A randomized, controlled trial of resistance training during hemodialysis. J Am Soc Nephrol 2007(18):1594-1601.
